# Evidence of pathogens associated with travelers’ diarrhea in Thailand: a systematic review

**DOI:** 10.1186/s40794-024-00243-y

**Published:** 2025-04-28

**Authors:** Wanida Mala, Kwuntida Uthaisar Kotepui, Frederick Ramirez Masangkay, Kinley Wangdi, Polrat Wilairatana, Manas Kotepui

**Affiliations:** 1 Medical Technology, Faculty of Science, Nakhon Phanom University, Nakhon Phanom, Thailand; 2 Department of Medical Technology, Faculty of Pharmacy, University of Santo Tomas, Manila, 1008 Philippines; 3 HEAL Global Research Centre, Health Research Institute, Faculty of Health, University of Canberra, Bruce, ACT 2617 Australia; 4 National Centre for Epidemiology and Population Health, Australian National University, Acton, ACT 2601 Australia; 5 Department of Clinical Tropical Medicine, Faculty of Tropical Medicine, Mahidol University, Bangkok, 10400 Thailand

**Keywords:** Travelers’ diarrhea, Systematic review, Thailand, Enterotoxigenic *Escherichia coli*, ETEC

## Abstract

**Background:**

Thailand, a major tourist destination, exhibits variations in sanitation and food safety practices that can lead to cases of travelers’ diarrhea (TD) caused by a plethora of pathogens. This systematic review synthesizes data on the pathogens associated with TD in Thailand, providing valuable insights into pathogen diversity and distribution, traveler profiles, and geographical regions of concern.

**Methods:**

This systematic review followed the PRISMA guidelines and was registered in PROSPERO (CRD42022346014). A comprehensive search was conducted across PubMed, Embase, Scopus, MEDLINE, and Journals@Ovid databases. The search included terms related to “diarrhea,” “travelers,” and “Thailand,” without restrictions on publication date. Eligible studies focused on travelers to Thailand who developed diarrhea with identified specific pathogens. Data was extracted and synthesized using a narrative approach. The risk of bias was assessed using the Joanna Briggs Institute (JBI) Critical Appraisal Checklist.

**Results:**

A total of 15 studies met the eligibility criteria, identifying that pathogens related to TD in Thailand were bacteria, particularly enterotoxigenic *Escherichia coli* (ETEC) (80%), followed by *Campylobacter jejuni* (33.3%) and *Salmonella* spp. (40%). Viral pathogens such as rotavirus and norovirus were also notable, with *Giardia* spp. being the most identified parasite. Pathogen distribution varied across different regions of Thailand, with tourism hubs such as Bangkok, Chiang Mai, Phuket, and Krabi reporting a broader range of infections.

**Conclusions:**

This systematic review highlights the diverse range of pathogens associated with TD in Thailand, with bacterial pathogens, specifically ETEC, being the predominant cause in most studies. The findings underscore the importance of preventive measures, such as improved hygiene practices and food safety awareness, especially in high-risk tourist areas. Further research is needed to understand better the risk factors contributing to TD and to develop targeted interventions for prevention.

**Supplementary Information:**

The online version contains supplementary material available at 10.1186/s40794-024-00243-y.

## Introduction

Travelers’ Diarrhea (TD) is the most frequent travel-associated disease experienced by international travelers to developing countries [1]. According to the Centers for Disease Control and Prevention (CDC), the attack rate of TD ranges from 10 to 70%, depending on the destination, season of travel and traveler characteristics [2]. Diarrhea cases have been defined as the passage of 3 or more unformed stools within a 24-hour period. TD is typically accompanied by symptoms such as nausea, vomiting, abdominal cramping, and fecal urgency [3, 4]. Recently, TD is classified based on scoring systems, which are determined by counting the frequency of loose stools [5]. A window of the first two weeks is typically used to determine the incidence rate of TD during the trip. The risk of acquiring TD is closely related to poor hygiene practices in local restaurants and inadequate hygiene and sanitation infrastructure [3]. Types of travelers may have an increased risk of TD, such as small children, younger travelers, backpackers, and volunteers [6]. Also, there have been reports that military personnel acquired infectious TD during military exercises [7].

The etiology of TD is primarily infectious, involving bacterial, viral, parasitic, or mixed pathogens. Bacterial infections are the most common cause of TD, accounting for an estimated 80–90% of cases globally. Enterotoxigenic *Escherichia coli* (ETEC) is the leading cause of TD, accounting for approximately 30–50% of cases [3, 6]. Other important pathogens include enteroaggregative *E. coli* (EAEC), *Shigella* spp., and *Campylobacter jejuni*, as well as various protozoa and viruses. Protozoan parasites account for a small percentage of TD cases, approximately 10%, while viral pathogens are thought to cause approximately 2–15% of cases [6, 8]. The ingestion of contaminated food or water is the main mode of transmission. It is most contracted in regions with lower hygiene and sanitation standards than the traveler’s home country, especially in parts of Africa, Latin America, and Southern and Southeast Asia [3, 6].

In Thailand, diarrhea remains a significant public health issue with notable variations across regions and seasons. The highest incidence rates are found in the central and northeastern regions [9, 10]. Seasonal peaks in cases occur during the winter (November to February) and the early rainy seasons (May to July), indicating that environmental factors influence transmission [9, 11]. The peak in winter is primarily caused by viral pathogens, such as rotavirus and norovirus, especially among young children. The peak during the early rainy season may be related to dysentery or other bacterial diarrhea [10, 12]. Vulnerable groups include children under 5 years old, who experience the highest rates of hospitalization and mortality [11]. With changing climate patterns, diarrhea is a persistent and potentially increasing health threat in the region. Thai healthcare addresses diarrhea through comprehensive case surveillance, prevention strategies, and effective treatment methods. Surveillance includes nationwide monitoring, outbreak investigations, and pathogen identification to inform appropriate responses [9, 13]. Prevention efforts focus on hygiene campaigns, ensuring food and water safety, and providing vaccinations. Treatment involves the use of oral rehydration therapy and the careful administration of antibiotics [14, 15].

Thailand, located in the center of mainland Southeast Asia, is known for its vibrant culture, breathtaking landscapes, and savory food, making it a popular destination for travelers worldwide. Thailand was the top regional tourist destination and experienced more than 10 million increase in foreign visitors [16, 17]. The number of overnight travelers in 2017 was 35.6 million [18]. However, like many tropical and subtropical regions, Thailand posed a risk for TD, a common health concern for visitors. In questionnaire-based surveillance studies, approximately 6–16% of foreign visitors reported having experienced TD while in Thailand [19–21]. A previous survey study found TD attack rates of 16% among Australians and New Zealanders, 8% among Europeans, and 7% among North Americans of 22,401 travelers departing from Phuket or Chiang Mai [19]. Another study found an incidence rate of 32.1% per person per month, mainly from Australia and New Zealand [20]. Moreover, a high prevalence of TD was reported among US military personnel participating in the previous Cobra Gold exercises in Thailand [22, 23]. *Campylobacter* spp. has been considered the most frequently isolated pathogen among Southeast Asian travelers [24–26]. In Thailand, *Campylobacter* spp., *Plesiomonas* spp., and *Vibrio* spp. were the most isolated pathogens from TD [1].

Although TD typically resolves independently within a few days without treatment and is unlikely to be fatal; this disease significantly impacts the quality of life and imposes an economic burden, including healthcare costs, travel change expenses, and changes to vacation or business plans [1, 27]. Given the lack of updated data to define the incidence of TD in Thailand, this systematic review aimed to synthesize data on the pathogens associated with TD in Thailand, providing valuable insights into pathogen distribution, traveler profiles, and geographical regions of concern.

## Methods

### Protocol registration

This systematic review was conducted following the Preferred Reporting Items for Systematic Reviews and Meta-Analyses (PRISMA) guidelines [28]. The protocol of the systematic review was registered at PROSPERO (CRD42022346014).

### Systematic review question and outcome

The Condition, Context, Population (CoCoPop) framework was applied to develop the systematic review question [29]. In this framework, the Condition was TD, the Context was Thailand, and the Population was travelers. The primary aim of the systematic review was to synthesize data on pathogens associated with TD in Thailand.

### Search strategy

A systematic search was conducted to identify studies relevant to the review topic, focusing on the epidemiology, causes, and impact of diarrhea among travelers to Thailand. The search strategy was designed using the CoCoPop framework to structure the review problem. Specifically, the target population was travelers, the condition of interest was diarrhea, and the geographical context was Thailand. Searches were performed across several electronic databases, including PubMed, Embase, Scopus, MEDLINE, and Journals@Ovid. The search terms were selected to encompass a broad range of relevant studies, combining synonyms and related terms for diarrhea, travel, and Thailand. The general search string was: (diarrhea OR diarrhoea OR dysentery OR “loose stool” OR “watery feces”) AND (travel OR traveler OR traveller OR tourism OR tourist) AND (Thailand OR Thai OR Siam) (Table S1). This search strategy allowed for flexibility in capturing different spellings, terminologies, and contexts. Only articles written in English were included, but there were no restrictions on the publication date of the studies, ensuring a comprehensive retrieval of relevant literature. The initial search was conducted on August 7, 2023, and updated on June 23, 2024.

### Eligibility criteria

Studies were included based on predefined inclusion and exclusion criteria. Studies were included if they (i) focused on travelers to Thailand who developed diarrhea, (ii) identified specific pathogens associated with the diarrhea, and (iii) were original research articles, including cohort studies, case-control studies, cross-sectional studies, and prospective observational studies. Studies were excluded if they (i) were case reports, reviews, or non-original research, (ii) were published in a language other than English without an available translation, or (iii) utilized the same dataset as another included study.

### Study selection and data extraction

After conducting the database searches, duplicate entries were removed using EndNote version 21.0 (Philadelphia, PA). Subsequently, the titles and abstracts of the remaining records were independently screened by two reviewers (WM, MK) to assess their relevance to the review’s objectives. Non-relevant records were excluded due to their irrelevance to the study population or outcomes of interest. The full texts of the remaining studies were then examined in detail, and those not meeting the eligibility criteria were excluded for specified reasons. Studies that met the eligibility criteria were included in the systematic review. Data from the included studies that met the inclusion criteria were extracted independently by two authors (WM, MK) using a standardized data extraction form. Discrepancies in study selection and data extraction were resolved through discussion, and a third reviewer (PW) was consulted if necessary. Key data extracted from each study included study design, year of publication, study location (single or multiple sites), travel purpose (e.g., tourism, military service, Peace Corps), origin of travelers, and the types and methods of pathogen identification. Data were categorized based on the type of pathogen (bacteria, viruses, protozoa) and the identification techniques employed, such as culture, polymerase chain reaction (PCR), enzyme immunoassay (EIA)/Enzyme-linked immunosorbent assay (ELISA).

### Risk of bias and data synthesis

The risk of bias in individual studies was assessed using the Joanna Briggs Institute (JBI) Critical Appraisal Checklist, tailored to each study design [30]. Two independent authors (WM, MK) conducted the risk of bias assessment. Any discrepancies were resolved through discussion or consulting a third reviewer (PW). A narrative synthesis was conducted to compile data on pathogens associated with TD in Thailand. The results were presented in frequency (percentage) and the geographical distribution of TD across the country.

## Results

### Study selection

**Fig. 1:**
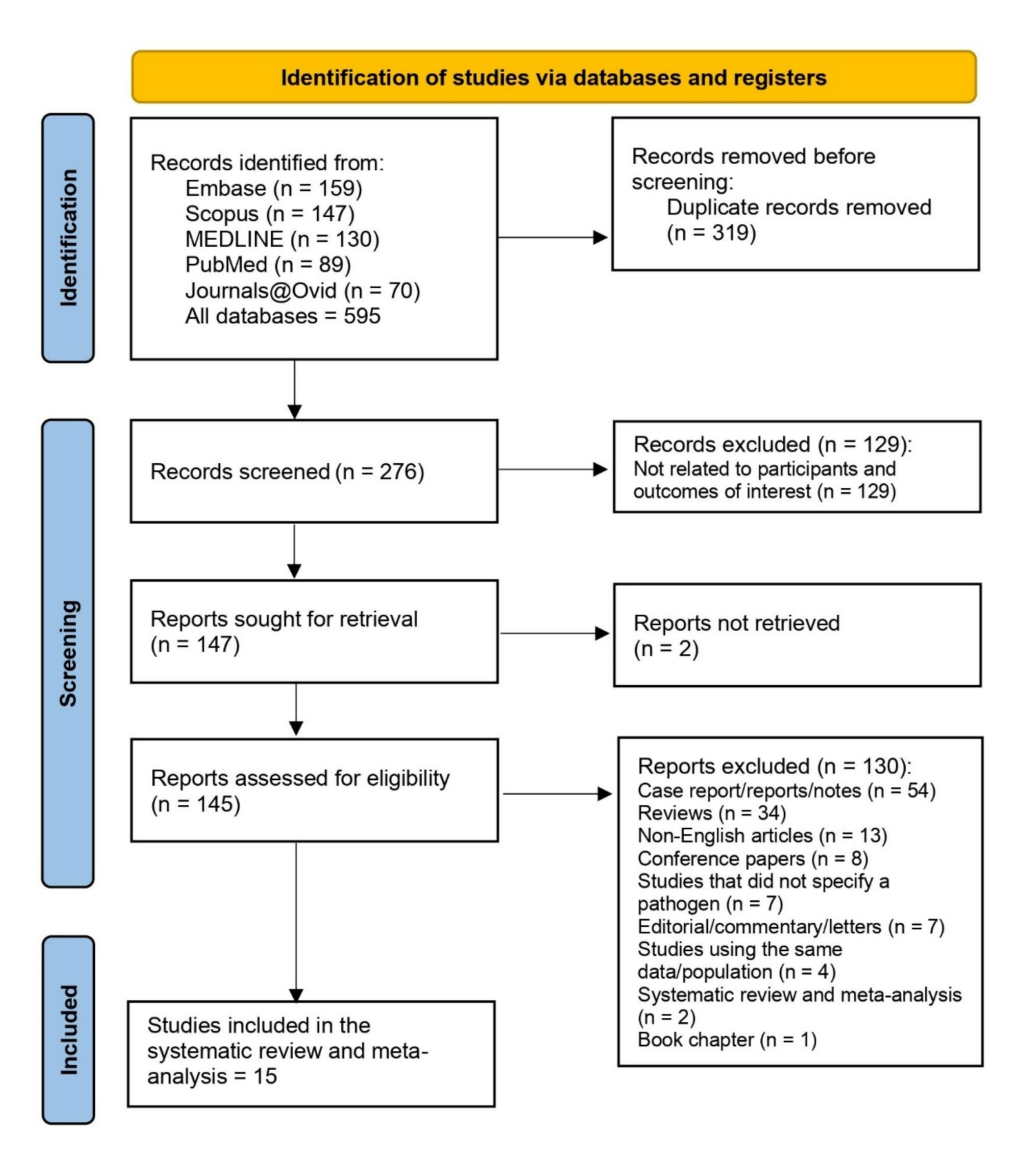
The PRISMA diagram illustrates the process of selecting studies for a systematic review

Initially, 595 records were identified from several databases, but after removing 319 duplicates, 276 records were screened. Of these, 129 were excluded for not being related to the study’s participants or outcomes of interest. The remaining 147 reports were further sought for retrieval, but two were not retrieved, leaving 145 for eligibility assessment. Out of these, 130 reports were excluded for reasons such as being case reports, reviews, non-English articles, studies that did not specify a pathogen, or using the same groups of participants. Finally, 15 studies met all criteria and were included in the final systematic review (Fig. 1). A meta-analysis was not performed due to significant variability and heterogeneity in the data from the included studies, and instead, a narrative synthesis was provided to summarize the findings comprehensively.

### Characteristics of included studies

Most of these studies were prospective observational studies (66.7%, *n* = 10), with others being case-control (20%, *n* = 3), and cross-sectional (13.3%, *n* = 2) (Table 1). The studies were published over several decades, with the majority released between 2010 and 2019 (33.3%, *n* = 5), followed by those from 2000 to 2009 (26.7%, *n* = 4) and smaller proportions from other periods. Nearly half of the studies were conducted across multiple sites (46.7%, *n* = 7), while others were either single-site studies (26.7%, *n* = 4) or did not specify the study site (26.7%, *n* = 4). The primary purpose of travel in these studies was tourism (46.7%, *n* = 7), with significant representation from US military personnel (40%, *n* = 6) and Peace Corps volunteers (20%, *n* = 3). Travelers primarily came from the United States (60%, *n* = 9), Oceania (26.7%, *n* = 4), Asia (26.7%, *n* = 4), Europe (26.7%, *n* = 4), North America (20%, *n* = 3), and Africa (6.7%, *n* = 1), while some studies did not specify the travelers’ origin (20%, *n* = 3).

**Table 1:** Characteristics of the 15 included studies

Characteristics	Number of studies (*n* = 15)	Percent (%)	References
**Study designs**			
Prospective observational studies	10	66.7	[4, 19, 31–38]
Cross-sectional studies	2	13.3	[22, 39]
Case-control studies	3	20.0	[1, 24, 40]
**Year of Publication**			
2020–2024	2	13.3	[4, 31]
2010–2019	5	33.3	[1, 24, 35, 37, 40]
2000–2009	4	26.7	[19, 22, 32, 38]
1990–1999	2	13.3	[34, 39]
1980–1989	2	13.3	[33, 36]
**Study sites**			
Single study site	4	26.7	[1, 24, 31, 36]
Multiple study sites	7	46.7	[4, 19, 34, 35, 38–40]
Not specified	4	26.7	[22, 32, 33, 37]
**Travel purpose**			
Tourism	7	46.7	[1, 19, 24, 31, 32, 35, 37]
US military personnel	6	40.0	[4, 22, 34, 35, 39, 40]
Peace Corps volunteers	3	20.0	[33, 36, 38]
**Origin/Nationality of travelers**			
United States	9	60.0	[4, 19, 22, 24, 33, 34, 36, 39, 40]
Oceania	4	26.7	[1, 19, 24, 31]
Asia	4	26.7	[1, 19, 24, 32]
North America	3	20.0	[1, 24, 31]
Europe	4	26.7	[1, 19, 24, 31]
Other	1	6.7	[19]
Not specified	3	20.0	[35, 37, 38]

### Risk of bias

For the two cross-sectional studies, both showed potential bias due to unclear strategies for addressing confounding factors [22, 39], and one study did not identify confounding factors [39]. For the case-control studies, all three met all 10 criteria, including comparability of groups, appropriate matching of cases and controls, use of standard and valid methods for exposure measurement and outcome assessment, identification and management of confounding factors, sufficient exposure period, and appropriate statistical analysis [1, 24, 40]. For the cohort/prospective observational studies, most demonstrated potential selection bias due to dissimilar groups or recruitment methods, while inconsistencies in exposure measurement indicated a risk of misclassification bias. Although confounding factors were often identified, only some studies adequately managed them [4, 33, 35]. Outcome measurement was generally valid and reliable, but several studies lacked strategies for handling incomplete follow-up [4, 32–34, 37] (Table S3).

### Characteristics of pathogens isolated from travelers

Half of the studies reported both single and multiple pathogens (66.7%, *n* = 10), while 26.7% (*n* = 4) focused on single pathogens, and 6.7% (*n* = 1) did not specify the pathogen type (Table 2). The most frequently identified pathogens were bacteria (40%, *n* = 6), with some studies detecting combinations of bacteria with viruses (33.3%, *n* = 5) or with protozoa and viruses (20%, *n* = 3). A few studies identified only protozoa (6.7%, *n* = 1). Various methods were used to identify pathogens, with culture/isolation and identification being the method for bacteria (100%, *n* = 15). Other bacterial identification methods included antimicrobial susceptibility testing (40%, *n* = 6), toxin production tests (33.3%, *n* = 5), PCR (20%, *n* = 3), real-time PCR (26.7%, *n* = 4), and serotyping (6.7%, *n* = 1). EIA/ELISA (46.7%, *n* = 7) and real-time reverse transcription PCR (40%, *n* = 6) were the main identification methods for viruses. In comparison, parasites were identified using microscopic examination and EIA/ELISA (33.3% each, *n* = 5), with real-time PCR used less frequently (13.3%, *n* = 2). Details of the pathogens associated with TD in Thailand are presented in Table S2.

**Table 2:** Characteristics of pathogens isolates from travelers

Characteristics	Number of studies (*n* = 15)	Percent (%)	References
**Pathogens**			
Single pathogen	4	26.7	[4, 19, 31–38]
Multiple pathogens	10	66.7	[1, 4, 19, 22, 24, 31, 36, 38–40]
Not specified (Single pathogen or multiple pathogens)	1	6.7	[33]
**Types**			
Bacteria	6	40.0	[19, 33–35, 37, 40]
Bacteria, virus	5	33.3	[4, 22, 31, 38, 39]
Bacteria, protozoa, virus	3	20.0	[1, 24, 36]
Protozoa	1	6.7	[32]
**Methods for identification of pathogens**			
**Bacteria**			
Culture/isolation and identification	15	100.0	[1, 4, 19, 22, 24, 31–40]
Antimicrobial susceptibility testing	6	40.0	[22, 31, 35, 37, 39, 40]
Toxin production test	5	33.3	[33, 34, 36, 38, 39]
PCR	3	20.0	[4, 24, 31]
Real-time PCR	4	26.7	[4, 24, 31, 37]
Serotyping by an agglutination assay	1	6.7	[35]
**Virus**			
EIA/ELISA	7	46.7	[4, 22, 33, 36, 38–40]
Real-time reverse transcription PCR	6	40.0	[1, 4, 22, 24, 36, 38]
**Parasite**			
Microscopic examination	5	33.3	[1, 32, 33, 36, 39]
EIA/ELISA	5	33.3	[1, 4, 19, 24, 40]
Real-time PCR	2	13.3	[4, 24]

Abbreviations: EIA, Enzyme immunoassays; ELISA, Enzyme-linked immunosorbent assay; Polymerase chain reaction; PCR, polymerase chain reaction

### The evidence of various pathogens associated with TD

Among the bacterial pathogens, *E. coli* strains were the most prevalent, particularly ETEC, found in 80% of the studies with 172 isolates (Table 3). Other *E. coli* strains, such as EPEC and EAEC, were also notable, found in 40% and 26.7% of studies, respectively. *C. jejuni* was another common bacterial pathogen identified in 33.3% of the studies (427 isolates). Bacteria belonging to the genus *Salmonella*, *Plesiomonas*, *Vibrio*, and *Aeromonas* were also frequently reported, appearing in 26.7–40% of the studies. Viral pathogens included rotavirus and norovirus, which were detected in 40% and 26.7% of studies. *Giardia* spp. was the most common among parasitic pathogens, found in 20% of the studies. Other less common pathogens, including *Arcobacter*, *Bacteroides fragilis*, *Clostridioides (Clostridium) difficile*, and *Helicobacter pylori*, were each identified in a small percentage of studies.

**Table 3:** The prevalence of various pathogens associated with TD across 15 studies

Pathogens	Number of studies (*n* = 15)	Percent (%)	Number of isolates/ samples	References
**Bacteria**				
***Campylobacter***				
*C. jejuni*	5	33.3	427	[4, 31, 35, 36, 40]
*Campylobacter* spp.	4	26.7	166	[1, 4, 24, 39]
*C. jejuni/coli*	4	26.7	80	[22, 33, 38, 40]
*C. coli*	1	6.7	10	[40]
***E. coli***				
ETEC	12	80.0	172	[4, 19, 22, 24, 31, 33, 34, 36, 38–40]
EPEC	6	40.0	100	[1, 4, 24, 31, 38, 40]
AEEC	1	6.7	28	[22]
EAEC	4	26.7	50	[4, 24, 31, 40]
ESBL-producing *E. coli*	1	6.7	8	[37]
EIEC	3	20.0	5	[1, 4, 24]
STEC	1	6.7	3	[24]
***Salmonella***				
*Salmonella* spp.	6	40.0	130	[1, 24, 31, 33, 38, 39]
Non-typhoidal *Salmonella*	2	13.3	67	[22, 40]
*S. enteritis*	1	6.7	10	[36]
***Plesiomonas***				
*Plesiomonas* spp.	4	26.7	100	[1, 22, 24, 39]
*P. shigelloides*	4	26.7	50	[4, 36, 38, 40]
***Vibrio***				
*Vibrio* spp.	4	26.7	67	[1, 4, 19, 24]
*V. parahaemolyticus*	4	26.7	20	[22, 36, 39, 40]
*V. cholerae*	1	6.7	2	[24]
*V. cholerae* non-O1	3	20.0	13	[22, 36, 38]
*V. cholerae* O1	1	6.7	1	[19]
*V. fluvialis*	1	6.7	1	[36]
***Aeromonas***				
*Aeromonas* spp.	6	40.0	48	[1, 4, 19, 24, 39, 40]
*A. hydrophila*	2	13.3	15	[33, 36]
***Shigella***				
*Shigella* spp.	6	40.0	40	[1, 4, 22, 24, 38, 39]
*S. flexneri*	1	6.7	4	[33]
*S. dysenteriae*	1	6.7	1	[33]
***Arcobacter***				
*Arcobacter* spp.	2	13.3	2	[1, 24]
*A. butzleri*	1	6.7	1	[4]
***Bacteroides fragilis***	1	6.7	11	[24]
***Clostridioides (Clostridium) difficile***	1	6.7	2	[24]
***Helicobacter pylori***	2	13.3	6	[4, 24]
**Virus**				
Rotavirus	6	40.0	24	[4, 22, 24, 36, 38, 39]
Norovirus	4	26.7	99	[1, 4, 24, 31]
Norwalk virus	2	13.3	14	[36, 38]
Sapovirus	1	6.7	6	[24]
Adenovirus	1	6.7	1	[40]
**Parasite**				
*Giardia* spp.	3	20.0	13	[1, 24, 39]
*Cryptosporidium* spp.	2	13.3	6	[1, 24]
*Blastocystis hominis*	1	6.7	2	[36]
*Capillaria* spp.	1	6.7	2	[32]
*Cyclospora* spp.	1	6.7	2	[24]

Abbreviations: ETEC, Enterotoxigenic *E. coli*; EPEC, Enteropathogenic *E. coli*; AEEC, Attaching and effacing *E. coli*; EAEC, Enteroaggregative *E. coli*; ESBL, Extended-spectrum beta-lactamase-producing *E. coli*; EIEC, Enteroinvasive *E. coli*; STEC, Shiga toxin-producing *E. coli*

**Fig. 2:**
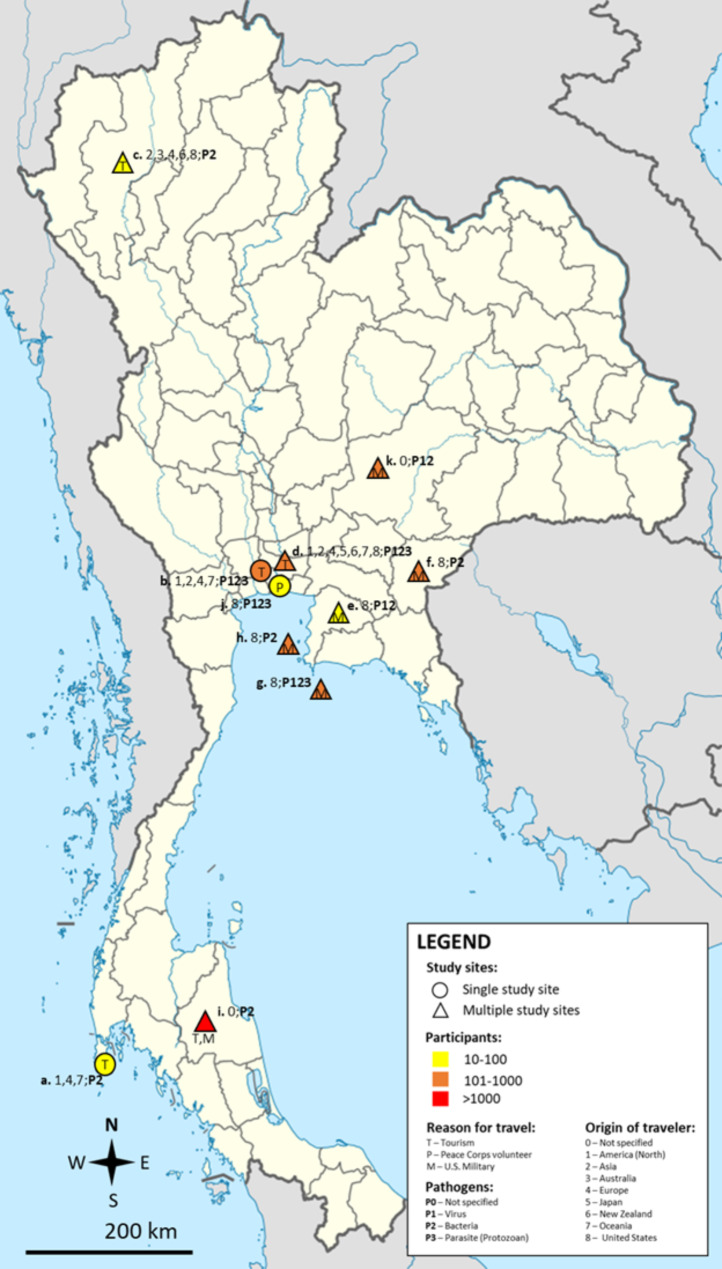
Geographic distribution of studies on diarrhea and pathogens among travelers in Thailand. **a**. Ashbaugh et al., 2020 [31]; **b**. Bodhidatta et al., 2019 [1]; **c**. Chongsuvivatwong et al., 2009 [19]; **d**. Lertsethtakarn et al., 2018 [24]; **e**. Lurchachaiwong et al., 2020 [4]; **f**. Mason et al., 2017 [40]; **g**. Petruccelli et al., 1992 [39] ; **h**. Serichantalergs et al., 1997 [34]; **i**. Serichantalergs et al., 2010 [35]; **j**. Taylor et al., 1985 [36]; **j**. Walz et al., 2001 [38]

The distribution of pathogens (bacteria, viruses, and parasites) and their association with different travel purposes were documented in different parts of the country (Fig. 2). For instance, regions with higher tourism activity, such as Bangkok, Chiang Mai, Phuket, and Nakhon Si Thammarat, seem to report a broader range of pathogens.

## Discussion

This systematic review demonstrated that pathogens related to TD in Thailand were bacteria, particularly ETEC, followed by *Campylobacter* (*C. jejuni*), and *Salmonella* spp. Viral pathogens such as norovirus and rotavirus were also notable. *Giardia* spp. was the most identified parasite. Additionally, the distribution of pathogens varied depending on the traveler’s purpose and the geographical region, with tourism hotspots such as Bangkok, Chiang Mai, Phuket, and Krabi reporting a broader range of pathogens. This aligns with previous reviews indicating that the incidence of TD varies depending on the traveler group and environment, with students and military personnel facing a moderately high risk [41]. Another systematic review also demonstrated that the destination, host factors, and duration of exposure among the environmental factors were the most critical risk factors for TD [42]. Moreover, the latest systematic review and meta-analysis identified younger age, extended travel durations, visits to low- and middle-income countries, backpacking travel styles, and pre-travel health conditions as risk factors for TD among individuals from high-income nations [5]. The influx of international tourists to these regions in Thailand might contribute to the increased diversity and prevalence of pathogens, possibly due to factors such as hygiene practices and contaminated food and drinks derived from unsanitary sources. Unsanitary preparation or contamination of foods and drinks are major contributors of TD [2]. Unsafe practices by food vendors, such as selling contaminated or overnight food, handling food without proper hygiene, and maintaining inadequate cleanliness around food stalls, significantly increase the risk of food contamination. Additionally, travelers increase the risk of TD by not washing their hands before meals, handling food with bare hands, and consuming undercooked or improperly prepared food [4, 20]. To address this issue, it is essential to improve sanitation, educate travelers about food safety, provide food handling training for vendors, and promote vaccinations and prophylactic measures. Implementing food handling courses has been shown to significantly reduce the risk of travelers developing TD [2, 43].

Research conducted in Thailand identified younger age, consuming street food, and not consistently washing hands after using the toilet as significant risk factors for the occurrence of TD [16, 20]. In addition, individuals traveling from low-transmission rural areas to higher-transmission urban centers within the same country are at an increased risk of contracting diarrhea -related illness [44]. In contrast, handwashing after toilet use and traveling with children or elderly persons were found to be significant protective factors, as people tend to adhere more to good hygienic practices when traveling with susceptible or vulnerable individuals [16]. Additionally, the origin of travelers was suggested to be a factor in the etiology of TD, as the etiologic agents of diarrhea vary by the traveler’s nationality, as observed in a previous study [1].

Several reviews reported bacterial, viral, and parasitic agents had caused TD [8, 45]. The present systematic review highlighted the significant burden of bacterial pathogens, particularly *E. coli* strains, which were the most frequently identified, with ETEC being predominant. This aligns with global trends where ETEC is a leading cause of TD, particularly in developing countries [46–48]. This also aligns with several reviews showing that most cases of TD are due to bacterial pathogens [8, 45]. The findings of this systematic review were consistent with those of previous reviews, which reported that most TD cases (62%) were caused by bacterial pathogens, particularly ETEC, EPEC, and *Campylobacter* [25]. Nevertheless, a previous review indicated that diarrhoeagenic *E. coli* (DEC) remains as TD’s most frequently implicated bacteria [6]. *Campylobacter* has traditionally been the leading cause of TD acquired during travel to Southeast Asia, including Thailand [1, 7]. The high prevalence of *Campylobacter* underscores the need for increased awareness and preventive measures, as this pathogen is commonly linked to foodborne illnesses and is known to cause severe gastrointestinal symptoms [49, 50]. US military personnel are frequently deployed to developing regions where enteric *Campylobacter* spp. that cause diarrheal disease are prevalent [1, 24].

Currently, reports of viral pathogens that caused TD are limited, as in this study. However, viral pathogens, including norovirus and rotavirus, were also significant contributors to TD, particularly in group settings such as military personnel and Peace Corps volunteers [31]. A cohort study found that norovirus is the most common cause of acute gastroenteritis among US and European travelers to high and moderate-risk areas [51]. Norovirus and rotavirus can induce cytopathic changes in the epithelial cells of the small intestine, leading to acute villous atrophy and a subsequent loss of enterocytes, which has been linked to a temporary decrease in disaccharidase activity and transient lactose intolerance during these infections [43]. The systematic review emphasizes the importance of considering viral agents in the differential diagnosis of TD, especially in cases where bacterial culture results are negative.

The identification of parasitic pathogens, though less frequent, highlights the ongoing risk of protozoan infections like *Giardia* spp., which can cause prolonged gastrointestinal symptoms [52, 53], and may be overlooked in routine diagnostic procedures. The presence of these pathogens in certain regions suggests that environmental factors, such as water quality and sanitation practices, play a crucial role in their transmission [54]. *Giardia* spp. can disrupt the epithelial brush border and intercellular adhesion, increase enterocyte apoptosis, and ultimately lead to enterocyte dysfunction, resulting in malabsorption and diarrhea [55, 56]. A previous review also indicated that giardiasis was the most common cause of infection-mediated persistent/chronic diarrhea in returning travelers [57]. *G. duodenalis* has also been reported as a cause of post-infectious irritable bowel syndrome in travelers returning from tropical or subtropical areas [58]. Similarly, *Blastocystis* spp. has been identified in patients suffering from gastrointestinal symptoms after returning from the tropics [59]. The human pathogenicity of *Blastocystis* spp. remains unclear. While most in vitro studies have demonstrated *Blastocystis* ST7 to induce cytopathic effects [60, 61], a recent study suggested *Blastocystis* ST4 may be a beneficial commensal [62].

The findings of this study are consistent with previous studies, which found EPEC, EAEC, ETEC, and *Campylobacter* as the most common pathogens, particularly in tropical and subtropical countries [31, 63, 64]. Similarly, a previous study examining British soldiers in Kenya found ETEC to be the most frequently detected pathogen [64]. However, there were notable differences in the pathogens identified in Nepal. In cases of TD, the most detected pathogens were norovirus, followed by ETEC, EPEC, *Campylobacter*, and EAEC [31]. Additionally, travelers returning from tropical and subtropical countries were always detected as co-infections with pathogens. Previous studies have reported that EAEC and EPEC are isolated more frequently in current TD cases [65, 66]. However, the symptoms of TD vary based on the causative pathogen. ETEC is usually associated with acute watery diarrhea. ETEC and EPEC may present similar clinical symptoms. In contrast, *Campylobacter* is often linked to more severe cases of TD, typically presenting with symptoms such as fever and abdominal pain [22, 67]. These findings highlight that the prevalence of a pathogen does not necessarily correlate with its clinical significance [66]. This emphasizes the need for comprehensive diagnostic and epidemiological studies to provide appropriate prevention and treatment strategies.

For all pathogens isolated from travelers, there were variations in the prevalence of several pathogens, which could have been influenced by the destination, season of travel, and the traveler’s nationality. The traveler’s origin has been considered to play a role in the etiology of diarrhea [1]. For example, *Campylobacter* was more prevalent among Europeans and North Americans, while *Salmonella* was more prevalent among Australians and New Zealanders [1]. On the other hand, there was heterogeneity in diagnostic methods for pathogens and variations in the rates of pathogen isolation. Diagnosing pathogens causing TD traditionally relied upon methods that could fail to detect in some cases [68, 69]. However, other techniques, such as PCR and real-time PCR have been increasingly used for diagnosing infections due to their high sensitivity, high specificity, and rapid turnaround compared with traditional diagnostic methods [69].

The systematic review findings demonstrate that regions with higher tourism activity, such as Bangkok, Chiang Mai, Phuket, and Krabi, report a broader range of pathogens. This diversity is likely due to several factors, including the high volume of international and domestic travelers, which increases the introduction and spread of various pathogens. Tourists in these areas are exposed to multiple sources of infection, such as local food, water activities, and crowded attractions, while environmental factors like warm and humid climates further support pathogen survival and transmission [70, 71]. Additionally, variability in sanitation, hygiene practices, and healthcare infrastructure across these regions may contribute to the observed range of pathogens.

This study has limitations. First, the inability to conduct a meta-analysis on the prevalence and risk of TD in Thailand due to significant variability in the data from the included studies limits the ability to quantify the overall burden of the disease. Additionally, a significant limitation is the potential for publication bias arising from excluding of unpublished data and grey literature, which might have led to an underrepresentation of certain findings. Second, there was a lack of standardization in pathogen identification methods across studies, with some failing to specify the diagnostic approaches used. This lack of consistency could affect the accuracy and comparability of the findings. The third limitation was there were TD reports from high tourist provinces in Thailand only. There were few reports of TD from the other parts of Thailand. Fourth, the studies included did not always provide detailed information on the pathogen subtypes or the specific geographic origins of the travelers, which limits the ability to assess region-specific risks and the impact of varying travel behaviors. Fifth, the including older studies, half of which were published before 2010, may not reflect current epidemiological trends, diagnostic advancements, or emerging pathogens associated with TD in Thailand. Changes in diagnostic methods, travel patterns, and public health measures over time could limit the applicability of older findings to the present.

Despite these limitations, the systematic review underscores the importance of ongoing surveillance and targeted interventions to reduce the risk of TD, particularly in high-risk areas of Thailand. Enhancing traveler education on safe food and water practices, particularly in high-risk areas. Also, considering prophylactic interventions is critical in mitigating the burden of TD. Future research should address this gap by using modern diagnostic techniques and focusing on current travel dynamics to understand the burden of TD better and guide targeted interventions. Additionally, ongoing research and public health efforts should focus on minimizing this common travel-related illness, particularly in at-risk populations and regions with high tourist influx.

## Conclusion

This systematic review provides a comprehensive overview of the pathogens contributing to TD in Thailand, highlighting the predominance of bacterial agents, particularly *E. coli* strains and *Campylobacter* spp., while acknowledging the role of viral and parasitic infections. The risk of TD was heterogeneous, with hotspots in Bangkok, Chiang Mai, Phuket, and Krabi. The findings underscore the need for ongoing and expanded research, improved diagnostic practices, and preventive strategies to protect travelers and reduce the burden of this common travel-related illness.

## Electronic supplementary material

Below is the link to the electronic supplementary material.

Supplementary Material 1

Supplementary Material 2

Supplementary Material 3

## Data Availability

No datasets were generated or analysed during the current study.
