# Supplementary material for: Evidence of pathogens associated with travelers’ diarrhea in Thailand: a systematic review
Source: Trop Dis Travel Med Vaccines. 2025 Apr 28;11:8. doi: 10.1186/s40794-024-00243-y (PMC12036122; doi:10.1186/s40794-024-00243-y)
Supplement: Supplementary file 1 — Supplementary Material 1 [file 40794_2024_243_MOESM1_ESM.docx]

**Table S1. Search term**

**General keywords**

(diarrhea OR diarrhoea OR dysentery OR “loose stool” OR “watery feces”) AND (travel OR traveler OR traveller OR tourism OR tourist) AND (Thailand OR Thai OR Siam)

PubMed 23 June 2024

| No. | Key concept | Search terms | Results |
| --- | --- | --- | --- |
| 1. | Diarrhea | diarrhea[Text Word] OR diarrhea[MeSH Terms] OR diarrhoea[Text Word] OR diarrhoea[MeSH Terms] OR dysentery[Text Word] OR dysentery[MeSH Terms] OR “loose stool”[Text Word] OR “loose stool”[MeSH Terms] | 156,033 |
| 2. | Travel | travel[Text Word] OR travel[MeSH Terms] OR traveler[Text Word] OR traveler[MeSH Terms] OR traveller[Text Word] OR traveller[MeSH Terms] OR tourism[Text Word] OR tourism[MeSH Terms] OR tourist[Text Word] OR tourist[MeSH Terms] | 77,822 |
| 3. | Thailand | Thailand[Text Word] OR Thailand[MeSH Terms] OR Thai[Text Word] OR Thai[MeSH Terms] OR Siam[Text Word] OR Siam[MeSH Terms] | 52,820 |
| 4. | 1 AND 2 AND 3 | #1 AND #2 AND #3 | 89 |

Embase 23 June 2024

| No. | Key concept | Search terms | Results |
| --- | --- | --- | --- |
| 1. | Diarrhea | diarrhea:ti,ab,kw,de OR 'diarrhea'/exp OR diarrhoea:ti,ab,kw,de OR 'diarrhoea'/exp OR dysentery:ti,ab,kw,de OR 'dysentery'/exp OR 'loose stool':ti,ab,kw,de OR 'loose stool'/exp | 398,679 |
| 2. | Travel | travel:ti,ab,kw,de OR 'travel'/exp OR traveler:ti,ab,kw,de OR 'traveler'/exp OR traveller:ti,ab,kw,de OR 'traveller'/exp OR tourism:ti,ab,kw,de OR 'tourism'/exp OR tourist:ti,ab,kw,de OR 'tourist'/exp | 113,819 |
| 3. | Thailand | Thailand:ti,ab,kw,de OR Thailand/exp OR Thai:ti,ab,kw,de OR Thai/exp OR Siam:ti,ab,kw,de OR Siam/exp | 66,234 |
| 4. | 1 AND 2 AND 3 | #1 AND #2 AND #3 | 159 |

Scopus 23 June 2024

| No. | Key concept | Search terms | Results |
| --- | --- | --- | --- |
| 1. | Diarrhea | TITLE-ABS-KEY ( diarrhea OR diarrhoea OR dysentery OR "loose stool" OR "watery feces" ) | 349,623 |
| 2. | Travel | TITLE-ABS-KEY ( travel OR traveler OR traveller OR tourism OR tourist ) | 482,958 |
| 3. | Thailand | TITLE-ABS-KEY ( diarrhea OR diarrhoea OR dysentery OR "loose stool" OR "watery feces" ) | 146,002 |
| 4. | 1 AND 2 AND 3 | 1 AND 2 AND 3 | 147 |

MEDLINE 23 June 2024

| No. | Key concept | Search terms | Results |
| --- | --- | --- | --- |
| 1. | Diarrhea AND Travel AND Thailand | (diarrhea OR diarrhoea OR dysentery OR “loose stool” OR “watery feces”) AND (travel OR traveler OR traveller OR tourism OR tourist) AND (Thailand OR Thai OR Siam) | 130 |

Ovid 23 June 2024

| No. | Key concept | Search terms | Results |
| --- | --- | --- | --- |
| 1. | Diarrhea AND Travel AND Thailand | (diarrhea OR diarrhoea OR dysentery OR "loose stool" OR "watery feces") AND (travel OR traveler OR traveller OR tourism OR tourist) AND (Thailand OR Thai OR Siam) {Including Limited Related Terms} | 70 |
